# Mining candidate genes for rice cadmium accumulation in the shoot through a genome-wide association study and transcriptomic analysis

**DOI:** 10.3389/fgene.2022.944529

**Published:** 2022-08-31

**Authors:** Jian Wang, Wu Yang, Shaohong Zhang, Jingfang Dong, Tifeng Yang, Yamei Ma, Lian Zhou, Jiansong Chen, Bin Liu, Junliang Zhao

**Affiliations:** Rice Research Institute, Guangdong Academy of Agricultural Sciences and Guangdong Key Laboratory of New Technology for Rice Breeding and Guangdong Rice Engineering Laboratory, Guangzhou, China

**Keywords:** rice, cadmium absorption, candidate gene, genome-wide association study, transcriptomic analysis

## Abstract

High cadmium (Cd) accumulation in rice is a serious threat to human health. The genetic mechanism of Cd accumulation in rice is highly complicated. To identify the low Cd accumulation in rice germplasm, investigate the genetic mechanism underlying Cd accumulation, and mine the elite genes of significant importance for rice breeding of low Cd accumulation varieties, we performed a genome-wide association study (GWAS) for rice Cd concentration in the shoot. The rice accessions were 315 diverse *indica* rice accessions selected from the 1568 rice accessions with 700,000 SNPs. Within the high rate of linkage disequilibrium (LD) decay, eight QTLs related to rice Cd accumulation were identified. Transcriptomic analysis showed there were 799 differentially expressed genes (DEGs) in the root and 857 DEGs in the shoot, which are probably considered to be the cause of the significant difference in Cd accumulation between high and low Cd accumulation varieties. In *qCd11-1,* we detected a crucial candidate gene, *LOC_Os11g11050,* which encodes an initiation factor, expressed differently in the root between the high and low Cd accumulation varieties. Furthermore, under Cd treatment, the expression levels of *LOC_Os11g11050* significantly decreased in both the high and low Cd accumulation varieties. Sequence comparison and qRT-PCR revealed that there were indel sequences and base substitutions in the promoter region of *LOC_Os11g11050* correlated with the *LOC_Os11g11050* expression level, as well as the phenotype of Cd concentration differences in shoot between the high and low Cd accumulation accessions. *LOC_Os11g11050* might play important roles in Cd accumulation. The results of our study provide valuable resources for low Cd accumulation in *indica* varieties and the candidate functional gene, as well as molecular mechanisms for Cd accumulation in *indica* rice. The genetic architecture underlying Cd accumulation in *indica* can be used for further applying the low Cd gene existing in *indica* for decreasing Cd accumulation in rice.

## Introduction

Cadmium (Cd) is one of the most dangerous heavy metals. Excessive intake of Cd may lead to severe health problems, including cancers of the lung, gallbladder, prostate, and urinary bladder (Nawrot et al., 2006). Rice tends to take up and accumulate a higher amount of Cd than other crops. As a staple food feeding almost half of the world population, efficient uptake of Cd by rice and transfer into the food chain pose a severe problem to human health and food safety (Clemens and Ma, 2016). In the past 3 decades, the rapid industrial development and lack of sufficient environmental protection have already resulted in widespread heavy metal pollution, and Cd contamination of rice has been reported in many major rice production countries, including China, India, Thailand, and Indonesia (Hu et al., 2016; Sui et al., 2018; Shi et al., 2020).

Treatments of soil, such as removal and replacement, chemical washing, or phytoremediation, can repair contaminated soil and reduce the Cd concentration of rice, but these methods may suffer from some disadvantages, such as high cost or time-consuming (Tang et al., 2017). Reducing the Cd concentration in rice by breeding low Cd accumulation varieties is a promising and cost-effective method to reduce the risks of Cd to human health without additional cost to farmers (Zhou et al., 2019).

Genetic variations contributing to lower Cd accumulation in rice are fundamental for breeding low Cd accumulation varieties. Fortunately, it had been reported Cd accumulation is genetically controlled in rice, and rice germplasm carries plenty of genetic variations related to low Cd accumulation (Rasheed et al., 2020). With the help of functional genetic tools (Gu et al., 2022; Sun et al., 2022), many genetic variations conferring low Cd accumulation in rice have been identified and functionally characterized, including *OsHMA3* (Ueno et al., 2010), *OsNRAMP5* (Sasaki et al., 2012; Tang et al., 2017), *OsNRAMP1* (Chang et al., 2020), *OsIRT1* (Lee and An, 2009), *OsIRT2* (Nakanishi et al., 2006), *OsCd1* (Yan et al., 2019), *OsHMA2* (Takahashi et al., 2012; Yamaji et al., 2013), *OsZIP7* (Tan et al., 2019), *OsCCX2* (Hao et al., 2018), *OsCAL1* (Luo et al., 2018), *OsLCT1* (Uraguchi et al., 2011), and *OsLCT2* (Tang et al., 2021). These have provided insights into the Cd accumulation mechanisms in rice and valuable genetic basics for breeding low Cd varieties.

However, most of the functional alleles or genetic variations related to low Cd accumulation were characterized by map-based cloning from *japonica* varieties, since *japonica* cultivars accumulated much lower concentrations of Cd than cultivars of other subpopulations (Li et al., 2017). Natural genetic variations contributing to low Cd accumulation in *indica* have not been reported, which greatly hampers the progress of breeding low Cd *indica* varieties, while the Cd contamination problem is much more severe in *indica* than *japonica*. So identifying low Cd accumulation accessions from *indica* germplasms and then characterizing genetic variations leading to low Cd accumulation, as well as dissecting molecular mechanisms underlying the high Cd accumulation in *indica*, offer a more practical and acceptable alternative way for breeding low Cd *indica* varieties.

In the previous study, we have successfully identified a few *indica* accessions with low Cd accumulation in grain from an international rice panel. Further GWAS analysis characterized a functional gene leading to a low Cd accumulation phenotype in *indica* (Zhao et al., 2018). These results indicated that highly diverse international germplasms are valuable resources in identifying low Cd accumulation *indica* accessions and then facilitate characterizing functional low Cd accumulation genes.

In the present study, we used the international rice panel to further identify *indica* accessions with low Cd accumulation in the shoot of the seedling stage. GWAS and whole-genome transcriptomic analysis were then conducted to characterize genes and mechanisms related to Cd accumulation in the shoot of the seedling stage. In the present study, we focused on Cd accumulation in the shoot of seedling, which is a combined indicator for Cd absorption by root and the following translocation from the root to the shoot. Generally, four key processes contribute to Cd accumulation in rice grains: 1) uptake and transport in roots, 2) translocation from the root to the shoot, 3) redistribution at nodes, and 4) remobilization to grains via the phloem (Hart et al., 2006; Uraguchi and Fujiwara, 2013; Huang et al., 2019). It has been reported that the reason for higher Cd concentration in *indica* than in *japonica* may be owing to the more efficient long-distance transport of Cd from the xylem to the shoot (Uraguchi and Fujiwara, 2013). Therefore, Cd translocation from the root to the shoot may be vital in determining Cd content differences between *indica* and *japonica*. The present study aimed to identify *indica* accessions with low Cd translocation and the functional genes conferring this phenotype in *indica*.

In order to address the aforementioned questions, in the present study, we focused on the shoot’s Cd content in the seedling stage under hydroponic culture using an international *indica* panel (McCouch et al., 2016), which include 315 diverse *indica* accessions from 45 countries. A genome-wide association study (GWAS) was then conducted for shoot Cd concentration with 700,000 single-nucleotide polymorphisms (SNPs) as the genotype. A total of 27 *indica* rice varieties with low Cd accumulation (< 20 mg/kg) in the shoot and eight QTLs related to low Cd accumulation were identified. One candidate gene *LOC_Os11g11050* for a significant QTL (*qCd11-1*) was predicted by combining results from GWAS, gene annotations, and transcriptomic analysis. This study provided valuable resources and a candidate functional gene for low Cd accumulation in *indica*, which are the basis for breeding of low Cd accumulation *indica* varieties. This study also dissected the molecular mechanisms underlying Cd accumulation in the shoot of the rice seedling stage, which provides novel insights into Cd accumulation in *indica* rice varieties.

## Results

### Phenotypic variations of the shoot’s cadmium concentration in 315 *indica* rice accessions

**FIGURE 1:**
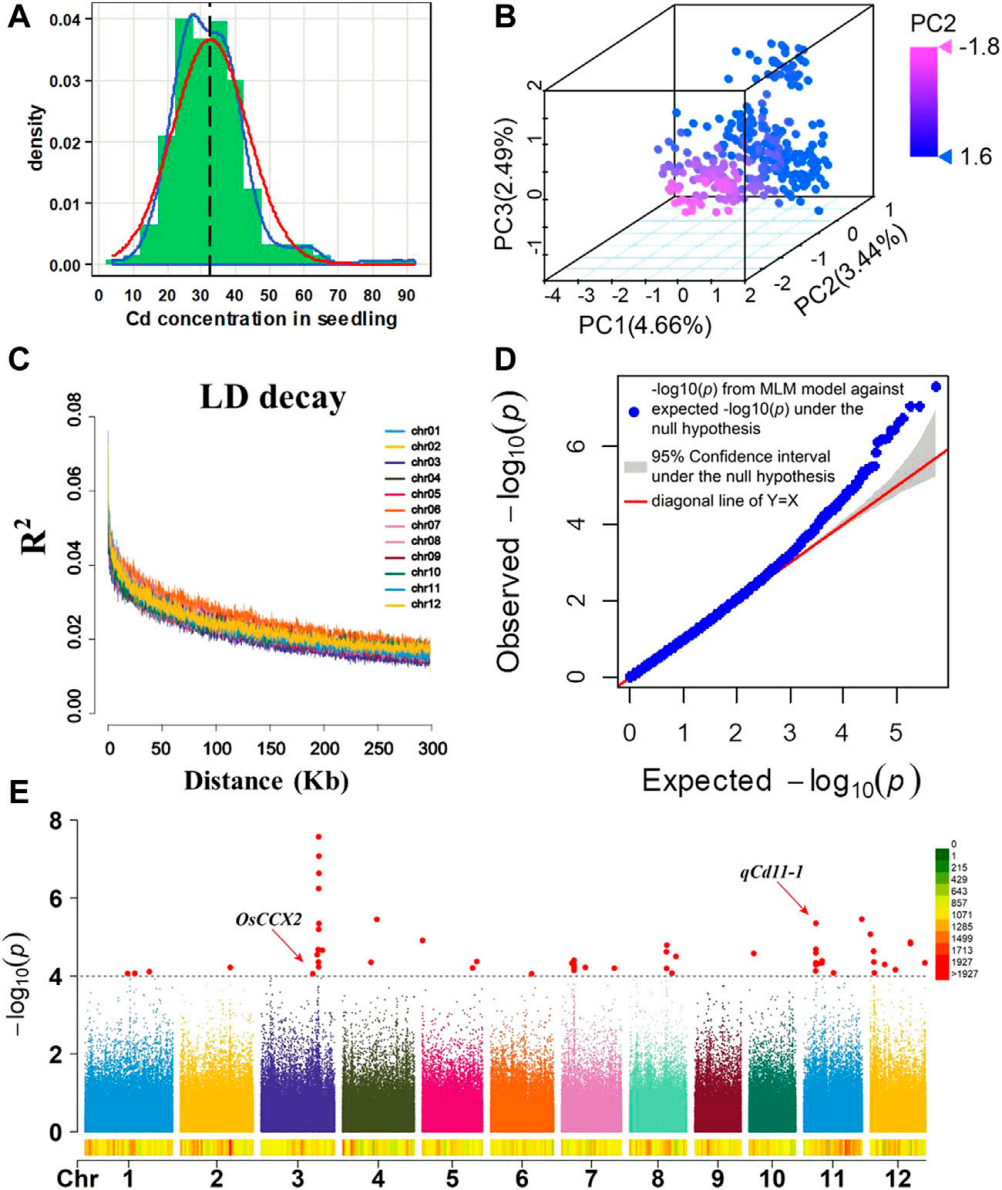
**(A)** Frequency distribution of the Cd concentration in 315 rice accessions. Blue line: trendline, red line: normal distribution line, black line: mean of the Cd concentration, and mean = 32.58. **(B)** Principal component analysis on 399,200 SNPs of 315 rice accessions. PC1, PC2, and PC3 represent the three principal components of the population. The color from red to blue represents the PC2 value. **(C)** Genome-wide average LD decay estimated in 315 rice accessions. **(D)** QQ plot for the GWAS of the Cd concentration in the shoot. *y*-axis: observed -log_10_(*p*) and *x*-axis: expected -log_10_(*p*) under the assumption that *p* follows a uniform[0,1] distribution. The red lines and gray region show the 95% confidence interval for the QQ plot under the null hypothesis of no association between the SNP and the trait. **(E)** Manhattan plots of the GWAS of shoot Cd accumulation in 12 chromosomes. The red arrow represents the loci close to previous genes. The gray dash line represents the significant threshold (*p* = 1.00 × 10^–4^).

A total of 315 diverse *indica* rice accessions from 45 countries were selected from an international panel with 1568 rice accessions (McCouch et al., 2016). Phenotypic analysis of the Cd concentration in the shoot of a seedling revealed a wide range of phenotypic variations among these accessions and approximately emerged on the normal distribution (Figure 1A). The Cd concentration for individual accession ranged from 4.07 to 92.14 mg/kg, with an average of 32.58 mg/kg. The *indica* cultivar “Cheriviruppu” from India had the lowest Cd concentration in the shoot, while the *indica* cultivar “P 660” from Pakistan had the highest Cd accumulation. In total, 27 rice accessions had Cd concentrations lower than 20.0 mg/kg (Table 1).

**TABLE 1:** Rice accessions with the Cd concentration lower than 20 mg/kg in the shoot.

Accession name	Subpopulation	Origin	Cd concentration in the shoot (mg/kg)
CHERIVIRUPPU	*indica*	India	4.07
WAS 200-B-B-1-1-1	*indica*	Senegal	10.42
EPAGRI 109	*indica*	Brazil	11.26
ICTA PAZOS	*indica*	Guatemala	13.49
ERH CHIANG TSAO 8	*indica*	China	13.88
KHAO DAW TAI	*indica*	Thailand	14.75
TSAKA	*indica*	Bhutan	15.44
E ZI 124	*indica*	China	15.62
UP 1537	*indica*	Colombia	15.65
RTS 5	*indica*	Vietnam	16.43
ARC 14500	*indica*	India	17.12
POONAGARI PERUMAL	*indica*	Sri Lanka	17.35
WAS 194-B-3-2-5	*indica*	Senegal	17.49
JARIYU	*indica*	India	17.71
EMBRAPA 6 CHUI	*indica*	Brazil	17.81
CIMARRON	*indica*	Venezuela	17.86
IR 70758-17-2-1	Admixed-*indica*	Philippines	17.93
JINLING 78-102	*indica*	China	18.52
CR 762022	*indica*	United States of America	18.63
DJOGOLON DJOGOLON	*indica*	Burkina Faso	18.73
WAS 207-B-B-3-1-1	*indica*	Senegal	19.06
VARY MADINIKA 3494	*indica*	Madagascar	19.65
JUMA 51	*indica*	Dominican Republic	19.71
ER MO ZHAN	Admixed-*indica*	China	19.8
IR 74371-3-1-1	*indica*	Philippines	19.8
MEKEO WHITE	*indica*	Papua New Guinea	19.84
BOL ZO	*indica*	Republic of Korea	19.97

### QTL mapping for cadmium accumulation in the shoot by a genome-wide association study

According to the criteria of minor allele frequency (MAF) being larger than 5% in the natural population, 399,200 SNPs were selected for GWAS from the 700,000 SNP dataset (McCouch et al., 2016). Principal component analysis (PCA) was performed with SNPs to estimate the population structure of these 315 rice accessions. Three distinct clusters were observed in the score plot of principal components (Figure 1B, Supplementary Figure S1). To enable visualization of the evolutionary history or relationship in the population, a phylogenetic analysis was performed. It showed that 315 rice accessions could be divided into three major clades (Supplementary Figure S2). In order to determine the rough region a QTL may span, we analyzed linkage disequilibrium (LD) decay on each chromosome in our GWAS population. LD analysis revealed that significant LD decays were observed at about 150–200 kb on all 12 chromosomes (Figure 1C). To control false positives by the population structure, a GWAS was conducted using a mixed linear model (MLM) model in GAPIT (Tang et al., 2016). The mixed linear model (MLM) is one of the most effective models which simultaneously incorporate both population structure and cryptic relationship (Yu et al., 2006). The quantile–quantile (QQ) plot was a useful tool for assessing how well the model used in the GWAS accounts for the population structure and familial relatedness. In the QQ plot (Figure 1D), the majority of the points lie on the diagonal line, which means most of the SNPs tested were probably not associated with the trait. The QQ plot results indicated that the false positive was well controlled in our GWAS analysis. It is expected that the SNPs on the upper right section of the graph deviate from the diagonal, which are most likely associated with the trait under study.

**FIGURE 2:**
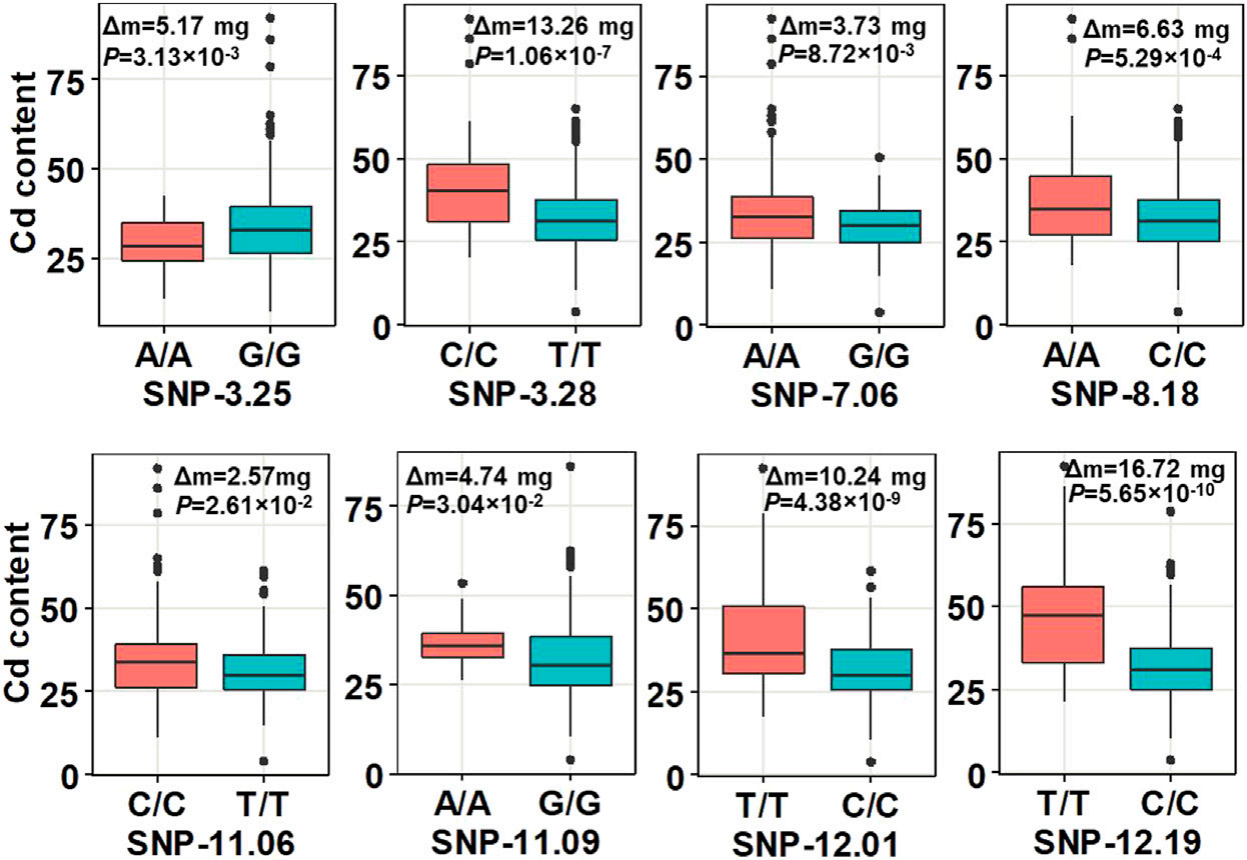
Boxplot of the phenotype analysis between the peak SNPs in the QTLs and phenotypic difference between minor alleles and major alleles. Δm, the difference of the mean of shoots’ Cd concentration between the minor alleles and major alleles at the seedling stage with three replications. Statistical comparison was performed by a one-sided *t*-test.

In summary, all the results from the abovementioned analysis demonstrated the reliability of our GWAS analysis. According to the LD decay results mentioned previously, a region was considered as one QTL where it had more than two SNPs with log_10_(*P*) >= 4 (FDR = 0.3) within a 200-kb window. The results of GWAS were shown using the Manhattan plot, which showed that eight QTLs with 27 SNPs were significantly associated with shoot Cd accumulation in the 315 *indica* rice accessions (Figure 1E). The SNPs with the highest significant signal on each chromosome are shown in Table 2. These QTLs (designated as *qCd* hereafter) are distributed on chromosomes 3, 7, 8, 11, and 12, which explained 4.36%–8.98% of the phenotypic variations. The MAF of the eight QTLs ranged from 0.07 to 0.38. The differences in the mean of shoots’ Cd concentration between the minor alleles and major alleles in the eight QTLs ranged from 2.57 to 13.26 mg (Figure 2).

**TABLE 2:** QTLs associated with Cd accumulation identified by the GWAS.

QTL	Chr	SNP	Allele	Position	MAF	*p*-value	FDR	Phenotype contribution (%)
*qCd3-1*	3	SNP-3.25	G/A	25,581,506	0.23	8.79E-05	0.30	4.36
*qCd3-2*	3	SNP-3.28	T/C	28,476,700	0.07	2.68E-08	0.01	8.98
*qCd7*	7	SNP-7.06	A/G	6,211,855	0.25	3.94E-05	0.25	4.8
*qCd8*	8	SNP-8.18	C/A	18,489,250	0.17	1.61E-05	0.18	5.3
*qCd11-1*	11	SNP-11.06	C/T	6,106,271	0.38	4.43E-06	0.10	6.02
*qCd11-2*	11	SNP-11.09	G/A	9,186,018	0.16	4.11E-05	0.25	4.77
*qCd12-1*	12	SNP-12.01	C/T	1,813,881	0.21	2.33E-05	0.21	5.09
*qCd12-2*	12	SNP-12.19	C/T	19,902,055	0.08	1.35E-05	0.17	5.39

### Whole-genome gene expression profile analysis between high- and low-cadmium accumulation accessions

In order to identify functional genes underlying QTLs, as well as dissect molecular mechanisms underlying Cd accumulation in the shoot of seedlings in *indica*, a whole genome expression profile was conducted by RNA-seq using roots and shoots from two high Cd accumulation accessions and two low Cd accumulation accessions. To detect whether the two high Cd content accessions have different gene expression patterns, we conducted a correlation analysis of the gene expression between the two high Cd accumulation accessions. The two high Cd accumulation accessions showed strong correlations of gene expression in the shoot (r > 0.95) and root (r > 0.85). The two low Cd accumulation accessions also showed strong correlations of gene expression in the shoot (r > 0.90) and root (r > 0.75) (Supplementary Figure S3). These results indicated the two high and two low Cd accumulation accessions have similar gene expression patterns.

Without Cd treatment (0 h), we identified 2,716 (1971 up- and 745 downregulated) differentially expressed genes (DEGs) in the root and 2047 (1430 up and 617 down-regulated) DEGs in the shoot between high and low Cd accumulation accessions (HR_LR_0 h for root samples; HS_LS_0 h for shoot samples). A total of 1423 (989 up- and 434 downregulated) DEGs in the root and 1424 (1013 up- and 411 downregulated) DEGs in the shoot were identified between the high and low Cd accumulation varieties under Cd treatment for 12 h (HR_LR_12h; HS_LS_12 h). Under Cd treatment for 48 h, 1327 (968 up and 359 down-regulated) DEGs in the root and 899 (604 up- and 295 downregulated) DEGs in the shoot were identified between the high and low Cd accumulation varieties (HR_LR_48h; HS_LS_48 h). The distribution patterns of DEGs were shown by the scatter plot (Supplementary Figure S4).

**FIGURE 3:**
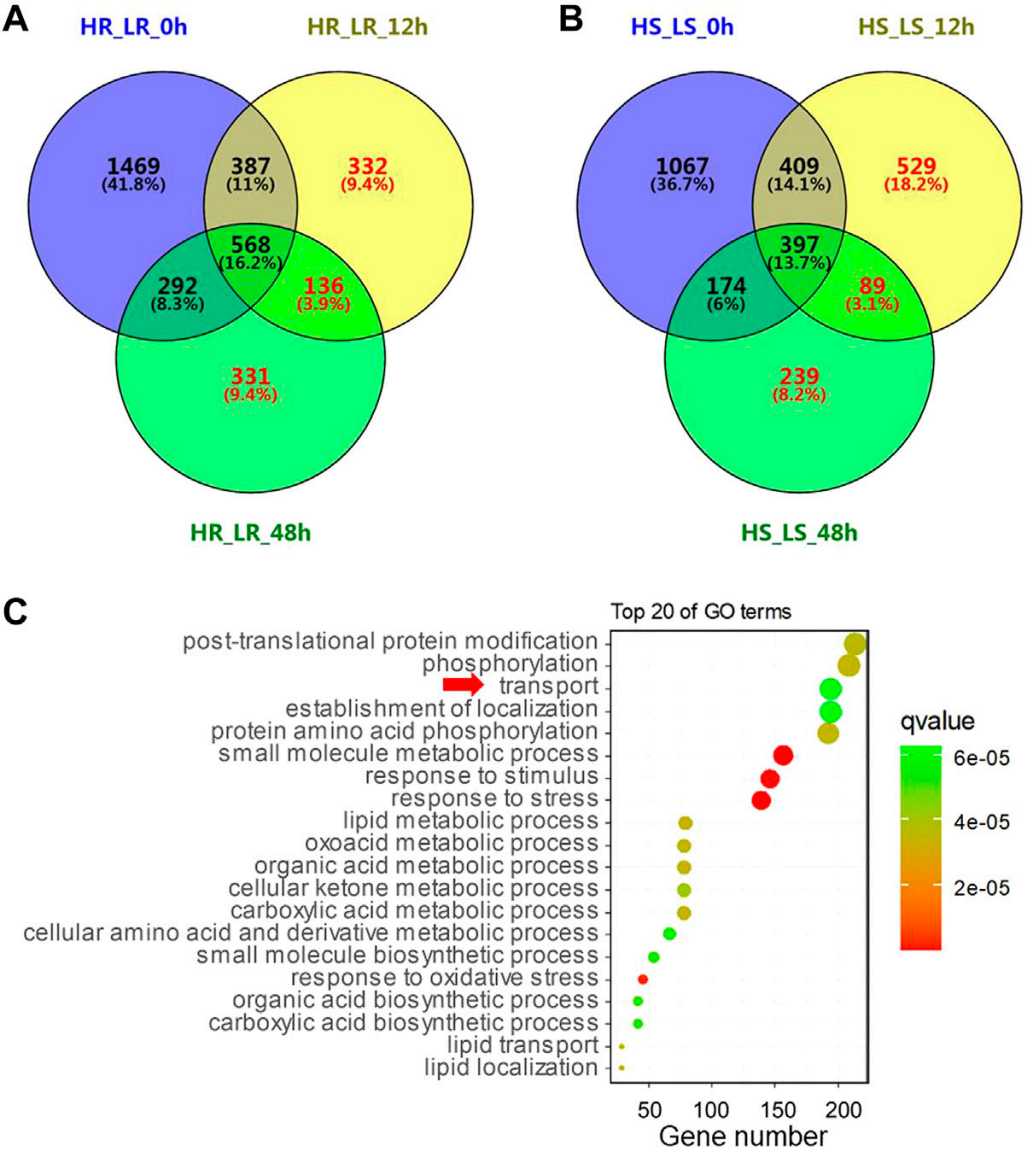
Analysis of differentially expressed genes (DEGs). **(A)** Venn diagram representing the number of DEGs between high and low Cd accumulation varieties in 0 h, 12, and 48 h after Cd treatment in the root. H and L represent two rice accessions with high Cd accumulation and two rice accessions with low Cd accumulation, respectively. R and S represent RNA extracted from the root and shoot, respectively. **(B)** Number of DEGs in the shoot. **(C)** GO enrichment of 20 important terms. The size of the circles represents gene numbers enriched in the GO terms.

In order to investigate the differential gene expression patterns between the high and low Cd accumulation accessions and the possible molecular pathways that responded to Cd treatment, Venn’s analysis was performed on six gene sets to obtain the subset of genes related to Cd accumulation differences between high and low Cd accessions (Figure 3). The results revealed that 799 DEGs in the root and 857 DEGs (region within red digital, in Figure 3) in the shoot responded to Cd treatment but were not included in DEGs of Cd non-treatment samples. These genes may be the functional genes related to Cd content differences.

To determine those genes which respond to Cd treatment, we compared the DEGs at 0 h with those at 12 h (or 48 h) with Cd treatment in the high Cd accumulation varieties and the low Cd accumulation varieties. Venn’s analysis (Supplementary Figure S5) was performed to reduce the influence on gene expression caused by different genetic backgrounds. We found 3,656 and 8,931 upregulated genes in the root and shoot, respectively, between control (no Cd treatment) and Cd treatment for 12h, containing 171 genes only in the root of the low Cd accumulation varieties (LR_0-12 h) and 450 genes only in the shoot of the low Cd accumulation varieties (LS_0-12 h). Meanwhile, 423 and 293 upregulated genes were found in LR_0-48 h and LS_0-48 h, respectively. Then, 113, 286, 383, and 341 downregulated genes were found in LR_0-12h, LS_0-12h, LR_0-48 h, and LS_0-48 h, respectively. The DEGs (the yellow region with red digital, in Supplementary Figure S5) which only exist in the low Cd accumulation varieties between control and Cd treatment are identified as genes probably related to lower Cd accumulation in the rice.

All the DEGs (red digital in Figure 3 and Supplementary Figure S5) were subjected to gene ontology analysis by agriGO v2.0 (Tian et al., 2017). The 10,853 DEGs were enriched in 105 GO terms, of which 64 were biological processes (BPs), three were cellular components (CCs) and 38 were molecular functions (MFs). The most significant enriched GO terms in BP were GO: 0006950 (response to stress), GO: 0044281 (the small molecule metabolic process), GO: 0050896 (response to stimulus), GO: 0006979 (response to oxidative stress), and GO: 0043436 (the oxoacid metabolic process). Interestingly, the “transport” (GO: 0006810) (Figure 3C) was among the most significant GO terms.

### Candidate gene identification in *qCd11-1*

Among the eight QTLs, *qCd3-2* had the most significant SNP at 28,476,700 in the region of 28.38-28.58 Mb on chromosome 3. Transcriptomic analysis results indicated only one gene (*LOC_Os03g50160)* showed a different expression pattern between roots from high and low Cd accumulation accessions (Supplementary Table S2). Furthermore, sequence difference analysis surrounding *LOC_Os03g50160* between high and low Cd accumulation accessions was conducted. However, no significant correlation was discovered between the sequence differences and Cd accumulation.

Interestingly, the *qCd3-1* identified from our GWAS results co-localized with a previously characterized gene (*OsCCX2*) that functioned in promoting upward transport of Cd in the xylem. Therefore, *OsCCX2* may be the candidate functional gene underlying *qCd3-1.* However, no expression differences were found between high and low Cd accumulation accessions in our transcriptomic results. Sequence comparison was also conducted in accessions of our GWAS population, but no SNPs or small indels were found in the coding region of *OsCCX2*. Further investigations are needed to characterize the functional variations of *OsCCX2* related to Cd accumulation in the natural population.

**FIGURE 4:**
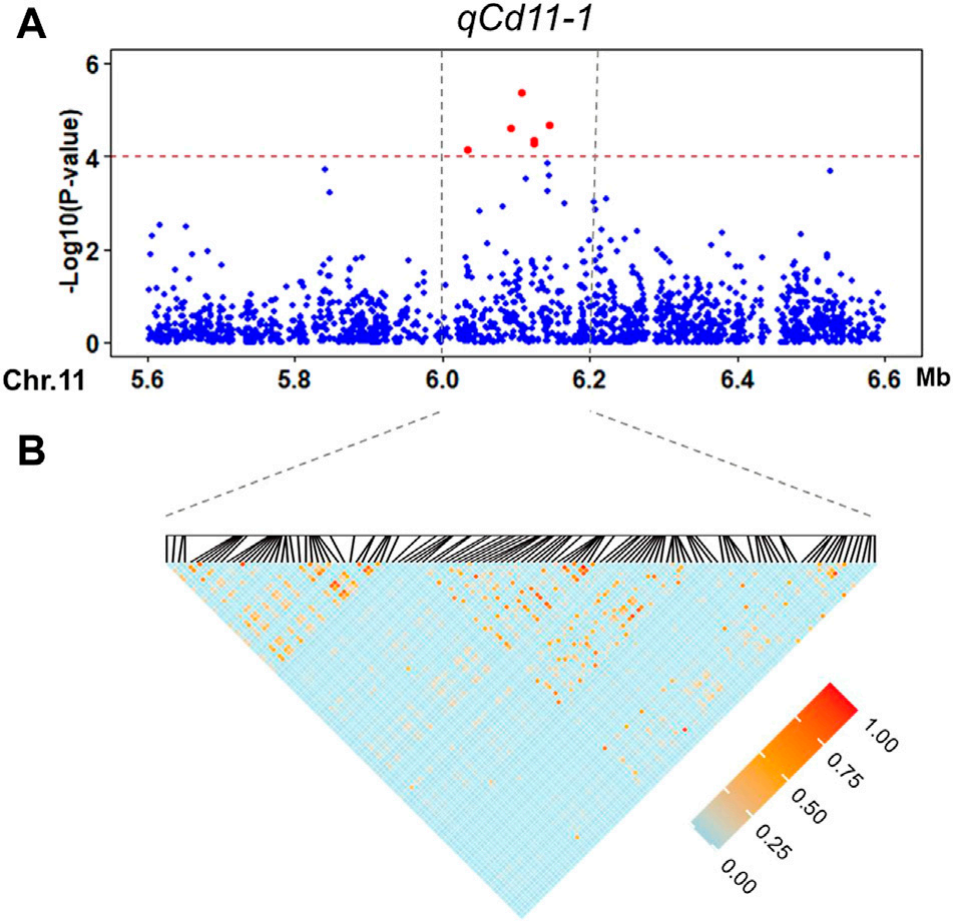
Candidate region estimation of *qCd11-1* on chromosome 11. **(A)** Local Manhattan plot of the GWAS for the Cd concentration in the shoot. **(B)** LD heatmap around the most significant SNP.

**FIGURE 5:**
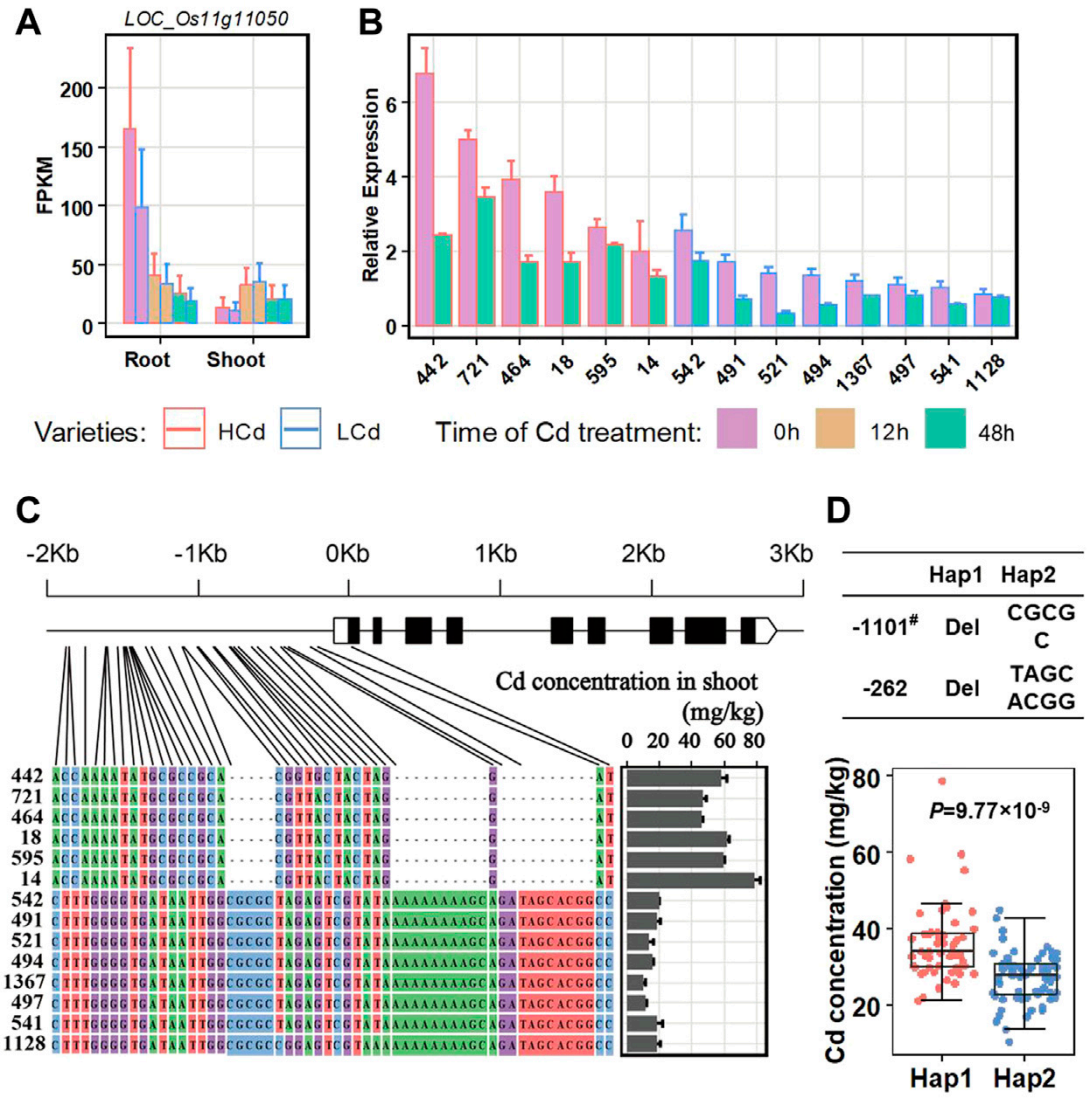
Expression changes of the candidate gene *LOC_Os11g11050* in the root and shoot after Cd treatment between high and low Cd accumulation varieties. **(A)** Detecting *LOC_Os11g11050* expression by transcriptomic analysis. **(B)** Detecting *LOC_Os11g11050* expression in the root with qRT-PCR. **(C)** Sequence comparisons of the *LOC_Os11g11050* promoter. HCd, high Cd accumulation varieties. LCd, low Cd accumulation varieties. **(D)** Boxplots for the Cd concentration based on haplotypes (Hap1 and Hap2; Hap1 had the deletions in the promoter, Hap2 did not have the deletions) of the *LOC_OS11g11050* promoter. ^#^ The deletion position is based on the initiator codon ATG of *LOC_OS11g11050*. Statistical comparison was performed by a one-sided *t*-test.

The second significant QTL in our GWAS result is *qCd11-1*, which has a large phenotype contribution (6.02%, Table 2) and a proper MAF (0.38), and may be one of the major QTLs controlling Cd accumulation in the *indica* panel. Candidate functional genes in *qCd11-1* were further analyzed in the present study. Then, results from transcriptomic analysis, gene annotation, and genome sequences analysis were combined to infer the candidate genes. The LD decay analysis in the *qCd11-1* interval delimited *qCd11-1* into an approximately 200-kb region (from 6.0 to 6.2 Mb on chromosome 11) (Figure 4). There were 31 genes annotated in the 200-kb region based on release 7 of the MSU Rice Genome Annotation Project (http://rice.uga.edu/). Transcriptomic analysis demonstrated that five genes were differentially expressed in the root or shoot between the high and low Cd accumulation accessions (Supplementary Table S2). Based on the expression pattern, *LOC_Os11g11050*, which encodes an initiation factor, was predicted to be the possible candidate gene of *qCd11-1* (Figure 5).

*LOC_Os11g11050* showed a relatively higher level of expression in the root than in shoot (Figure 5A). In the root, *LOC_Os11g11050* had a relatively higher level of expression in the high Cd accumulation accessions than the low Cd accumulation varieties. Under Cd treatment, the expression levels of *LOC_Os11g11050* significantly decreased in both the high and low Cd accumulation accessions. The results were further confirmed by qRT-PCR assays with six rice accessions with high Cd accumulation and eight rice accessions with low Cd accumulation (Figure 5B; Table 3).

**TABLE 3:** High and low Cd accumulation varieties for qRT-PCR and sequence analysis.

SEQ	Accession name	Subpopulation	Origin	Cd concentrationin shoot (mg/kg)
442	BADA DHAN	*indica*	Bangladesh	58.16
721	GEETA	*indica*	India	46.84
464	CCT 3-37-3-3-3-1	*indica*	Philippines	45.93
18	CO 25	*indica*	India	61.44
595	MOTTA SAMBA	*indica*	Sri Lanka	59.61
14	CHITRAJ (DA 23)	*indica*	Bangladesh	78.79
542	JUMA 51	*indica*	Dominican Republic	19.71
491	DJOGOLON DJOGOLON	*indica*	Burkina Faso	18.73
521	ICTA PAZOS	*indica*	Guatemala	13.49
494	E ZI 124	*indica*	China	15.62
1367	WAS 200-B-B-1-1-1	*indica*	Senegal	10.42
497	EPAGRI 109	*indica*	Brazil	11.26
541	JINLING 78-102	*indica*	China	18.52
1128	CR 762022	*indica*	United States of America	18.63

Sequence comparisons between the aforementioned accessions with contract Cd accumulation phenotypes and *LOC_Os11g11050* expression patterns were further investigated by sequencing of PCR production to identify whether there are sequential differences leading to the differences between Cd accumulation and the expression of *LOC_Os11g11050*. The results revealed that there were a few indels and base substitutions in the promoter region of *LOC_Os11g11050* between the high and low Cd accumulation accessions (Figure 5C), which constitute two haplotypes. The haplotypes showed a strong correlation with the expression level of *LOC_Os11g11050* and the Cd concentrations in the shoot (Figures 5B,C). The correlation between the haplotypes and Cd concentration was further investigated in the whole GWAS panel. The results showed accessions with Hap1 (with a deletion in the promoter) had more Cd concentration in the shoot than the accessions with Hap2 (without deletion in promoter) (Figure 5D). It seemed that the transformation of the *LOC_Os11g11050* promoter sequence resulted in a change in the gene expression and further altered the phenotype of Cd concentration in the shoot. *LOC_Os11g11050* might be a possible candidate gene for *qCd11-1*.

## Discussion

Cd accumulation in rice poses a severe risk to human health. *Indica* varieties generally accumulate higher Cd than *japonica*. So for most of the Cd-related QTLs or functional genes, the favorable alleles for reducing Cd accumulation were mainly derived from *japonica* rice varieties, which limited their breeding application in *indica* rice varieties. Since *indica* varieties are mainly planted in South China and Southeast Asia, where the Cd pollution problem is relatively more severe, it is of urgent need to identify low Cd accumulation accessions, as well as functional genes controlling Cd accumulation derived from *indica* germplasm.

In the previous study, we successfully identified *indica* accessions with low Cd accumulation in grains using an international diverse panel (Zhao et al., 2018). These results not only implied natural genetic variations controlling low Cd accumulation may exist in *indica* germplasm but also highlight the importance of utilizing highly diverse germplasm in screening and identifying these variances from *indica*. This is extremely important for Cd accumulation investigation and breeding in *indica*.

In the present study, we focused on the Cd accumulation in the aerial part of rice seedlings, which is determined by both Cd uptake by the root and the following transfer from the root to the aerial part. Previous studies had indicated these two factors are the key factors determining the Cd accumulation phenotype variations between *indica* and *japonica* (Chen et al., 2019; Liu et al., 2020). Therefore, we used a diverse rice collection consisting of 315 international *indica* rice accessions as materials, which represents an excellent resource for genetic diversity covering a wide geographical variation, and then facilitated natural genetic variation characterization. Furthermore, in the present study, the Cd content in the aerial part was assayed in seedlings treated under hydroponic culture conditions. Hydroponic culture can provide a uniform condition for Cd accumulation assays. All three replicates of 315 accessions were treated in a water pool by regular stirring, which provide a constant and uniform Cd concentration for all seedling samples. The phenotype results in the present study clearly demonstrated a wide range of Cd content in the aerial part of seedlings among 315 *Indica* rice accessions, which ranged from 4.07 mg/kg to 92.14 mg/kg, which further proved our previous assumption that many natural genetic variations controlling low Cd accumulation exist in *indica* germplasm. From the results, a few low Cd *indica* accessions were successfully identified from our diverse panel, which may be valuable for future breeding of low Cd *indica* varieties.

GWAS was then conducted using the Cd accumulation as the phenotype and the 700-K SNP dataset as the genotype. A total of eight QTLs related to Cd accumulation in the shoot of seedlings were identified by the GWAS. Chromosomal position comparisons revealed that *qCd3-1* co-localized with *OsCCX2*, a gene encoding a putative transporter, which had been identified to participate in root-to-shoot translocation of Cd in rice (Figure 1E). It has been reported that *OsCCX2* can promote an upward transport of Cd in the xylem. Knockout of the *OsCCX2* gene can reduce the transfer rate of Cd from roots to the aerial organs (Hao et al., 2018). Therefore, *OsCCX2* may be the candidate gene underlying *qCd3-1*. These results indicated the reliability of the GWAS results of this study. However, no expression pattern differences and small variations were found in *OsCCX2* in our GWAS panel. Further investigations are needed to further identify if there are structural variations in *OsCCX2* in the natural population, which may be related to Cd accumulation in rice.

Interestingly, we also found one QTL (*qCd8*) identified in the present study co-localized with another QTL (*qCd8*-2) controlling grain Cd accumulation in our previous study (Zhao et al., 2018). These results demonstrated this QTL may function both in Cd accumulation of the shoot and grain.

We found that most of the QTLs in the present study are novel QTLs related to Cd accumulation. There were three unique characteristics of GWAS in the present study for contributing to the discovery of novel QTLs. First, all 315 accessions in the GWAS population were *indica,* which were highly diverse international germplasm. The phenotypic distribution showed the *indica* population has a large range of Cd accumulation. Most of the previous studies were focused on populations containing both *indica* and *japonica* rice accessions, which may readily characterize QTLs controlling Cd accumulation differences between *indica* and *japonica*. Here, while using all *indica* varieties as the GWAS population, the specific genetic variations controlling low Cd accumulation with *indica* are possibly identified. Second, all accessions grew in a uniform hydroponic culture condition. The environmental variance in hydroponic culture conditions was less than that in the paddy field environment. Also, it would facilitate more accurate phenotyping. Third, we focused on the Cd concentration of the shoot at the seedling stage and used it as a phenotype for the GWAS, which was less investigated in previous studies. The phenotype ensured identification of QTL controlling both Cd absorbed by the root and Cd translocation from the root to shoot as well. It has been reported that the more efficient long-distance transport of Cd from the xylem to the shoot may be essential for higher Cd concentration in *indica* than in *japonica* (Uraguchi and Fujiwara, 2013).

In the present study, we were able to identify a candidate functional gene, *LOC_Os11g11050,* for *qCd11-1* by combining the GWAS and whole genome expression profile. The most significant SNP of *qCd11-1* locates 632 bp downstream from the candidate gene *LOC_Os11g11050.* Transcriptomic analysis demonstrated *LOC_Os11g11050* was expressed differently in the root between the high and low Cd accumulation varieties. Moreover, *LOC_Os11g11050* expression showed a significant response to Cd treatment. The qRT-PCR assays with six high Cd accumulation varieties and eight low Cd accumulation varieties further confirmed *LOC_Os11g11050* had a relatively higher level of expression in the high Cd accumulation varieties than in the low Cd accumulation varieties. Under Cd treatment, the expression levels of *LOC_Os11g11050* significantly decreased in both the high and low Cd accumulation varieties. *LOC_Os11g11050* encoded an initiation factor 2 subunit family domain-containing protein. It may initiate other Cd translocation genes’ expression in the root for the transport of Cd from the root to shoot. The sequence comparison of *LOC_Os11g11050* between high and low Cd accumulation varieties indicated that there were a few indels and base substitutions in the promoter region of *LOC_Os11g11050* between the high and low Cd accumulation varieties and constitute two haplotypes (Hap1 and Hap2). The haplotypes strongly correlated with the expression level of *LOC_Os11g11050* and the phenotype of the Cd concentration in the shoot. The *indica* accessions harboring Hap2 in the *LOC_Os11g11050* promoter had a lower Cd concentration than the *indica* accessions with Hap1. We thus regarded Hap2 in the *LOC_Os11g11050* promoter as a favor haplotype contributing to lower Cd accumulation in the shoot in *indica* rice. Applying the haplotype for marker-assisted breeding may have a potential application value in low Cd *indica* cultivars.

In conclusion, the present study provided a genetic analysis of Cd accumulation in the shoot within *indica* germplasm. Our results highlight the importance of diverse germplasm in studying Cd accumulation in rice, especially for *indica*. Using the international diverse *indica* panel and state-of-the-art functional genomic methods, we were able to identify novel QTLs and the underlying candidate gene for low Cd accumulation in the shoot of the seedling stage. These results provided novel resources, QTL, candidate genes, and molecular markers, which are essential for breeding low Cd *indica* varieties. The present study also provided novel insights into Cd transfer and accumulation in the aerial part of rice seedlings, which may be valuable for future studies on molecular mechanisms underlying Cd accumulation in rice.

## Materials and methods

### Rice accessions

A total of 315 rice *Indica* accessions (Supplementary Table S1) from 45 countries were selected according to the 1568 diverse rice accessions based on their 700,000 SNP genotypes and their origins (McCouch et al., 2016). Seeds of all 315 lines were provided by the International Rice Research Institute (IRRI).

### Sampling and cadmium detection

All 315 accessions were germinated and planted in a cultivation pot treated with 1.8 mg/kg Cd at the greenhouse for a month. Each line had three replicates. To determine the shoot Cd concentrations of the 315 rice accessions, the shoots of 10 seedlings in each replicate were pooled and dried in an oven at 70°C for 24 h. Then, the dried shoot was ground into powder and digested with an acid mixture of HNO_3_–HClO_4_. The Cd concentration was determined by inductively coupled plasma optical emission spectrometry (ICP-OES, iCAP6000, Thermo Scientific, United States).

### Genotyping, population structure, and genome-wide association study

GAPIT version 2 was used for GWAS analysis (Tang et al., 2016). The raw SNPs were exactly the same as the 700 K assay of a previous study (McCouch et al., 2016). The SNPs were selected for GWAS analysis by the criteria of having less than 15% missing data and minor allele frequency (MAF) > 0.05. The GWAS was conducted using the mix linear model (MLM) with the kinship matrix, and PC was set to three in GAPIT. We set the parameter “model. selection = Ture,” so GAPIT can find the optimal number of PC from 0 to 3. Considering the high complexity of the Cd accumulation mechanism and the specification of materials and tissue in the present study, we adopted a threshold *p* = 10^–4^ (false discovery rate = 0.3) at the genome-wide level. Manhattan and QQ plots were produced using the R package CMplot.

The rice genome sequence version of MSU V7.0 was used as a reference for analysis (Kawahara et al., 2013). We follow the criteria of having one associated locus between any two significant SNPs within a 200-kb interval. After determining the QTLs of GWAS analysis, the candidate genes were searched from 200 kb upstream and downstream of the most significant SNP in each QTL. All the genes located in the QTL region were predicted by the Rice Genome Annotation Project (MSU-RGAP, Nipponbare version 6.1).

### Transcriptomic analysis

We defined the varieties with shoot Cd concentrations less than 20 mg/kg as low Cd accumulation varieties (top 10% of the varieties with extreme low Cd phenotypes) and the varieties with shoot Cd concentrations of more than 45 mg/kg as high Cd accumulation varieties (top 10% of the varieties with extreme high Cd phenotypes). We chose two varieties (Supplementary Table S1, SEQ: 1294 and 517) from the high Cd accumulation variety group and two varieties (Supplementary Table S1, SEQ: 1154 and 808) from the low Cd accumulation variety group by random for transcriptome analysis. All samples were germinated and planted in a cultivation pot without Cd at the greenhouse. Three weeks later, the samples were treated with CdCl_2_. Two biological RNA replicates of the shoot and root under 1 μmol/L CdCl_2_ for 0, 12, and 48 h were extracted with the RNeasy Kit (AiDeLai, China). The RNA samples were evaluated on agarose gels, quantified in a spectrophotometer, and stored at −80°C. The RNA samples were then sequenced using a HiSeq-2500 instrument, and 10 Gb of raw sequencing data were obtained. The raw RNA-seq reads were initially processed to remove the adapter sequences and low-quality bases with Trimmomatic version 0.33 (Bolger et al., 2014) in the paired-end mode with recommended parameters. The virus-like and rRNA-like RNA-seq reads were further removed with fastq_clean (Zhang et al., 2014). Finally, the clean RNA-seq reads were mapped to the reference genomes using STAR (Dobin and Gingeras, 2015) version 2.5.0b. To improve spliced alignment, STAR was provided with exon junction coordinates from the reference annotations. The parameters ‘–runMode alignReads –twopassMode Basic –outSAMstrandField intronMotif –outFilterMultimapNmax 1 –genomeDir GenomeIndex –sjdbGTFfile Msu70.gft –alignIntronMax 30,000 –sjdbOverhang 100 –outSAMattributes All –outSAMattrIHstart 0 –outSAMtype BAM SortedByCoordinate –quantMode GeneCounts’ were used, and the outSAMattrIHstart parameter was changed to 0 for compatibility with downstream software Cufflinks. Strong correlations (r > 0.95) of gene expression were detected in the biologically replicated samples. Gene expression was measured using Cufflinks and cuffdiff2 (Trapnell et al., 2013) with the parameters‘–library-norm-method classic–fpkm –emit-count-tables –L label1,label2 Msu70.gtf sample1.rep1.cxb, sample1.rep2.cxb sample2.rep1.cxb, and sample2.rep2.cxb’. Fragments per kilo-base of exon per million fragments mapped (FPKM) were obtained. Genes with low expression values (FPKM < 1) were filtered for downstream analysis. The rice genome sequence version of MSU V7.0 was used as a reference. Genes that were differentially expressed between the two high Cd accumulation varieties and the two low Cd accumulation varieties were identified based on their corrected *p*-values. Gene ontology (GO) analysis was performed through agriGO2 (http://systemsbiology.cau.edu.cn/agriGOv2/).

### Differential expression analysis of genes by qRT-PCR

RNA reverse transcription reactions were performed using the PrimeScript ^TM^ RT reagent kit (TaKaRa, Japan). The primers for qRT-PCR were designed by Primer Premier 3.0. The ubiquitin was used as endogenous normalized genes for mRNA. Real-time PCR was carried out using the SYBR Premix ExTaq TM kit (TaKaRa, Japan) on a Bio-Rad CFX 96 Real-Time System. All reactions were run in triplicate. Primers used to amplify the selected genes are listed in Supplementary Table S3.

### Haplotype analysis

The leaves of rice seedlings were collected and subjected to DNA extraction by the CTAB method. The primers for gene *LOC_Os11g11050* PCR amplification are listed in Supplementary Table S3. The productions of PCR were sequenced, and the sequences were assembled by the software codon code at (https://www.codoncode.com/aligner/).

### Data analysis

The phylogenetic tree was constructed by MEGA 7.0 using the SNP abovementioned data. A *t*-test was conducted using Excel to detect the significant differences in gene expressions of high and low Cd accumulation varieties.

## Data Availability

The datasets presented in this study can be found in online repositories. The names of the repository/repositories and accession number(s) can be found at: https://www.ncbi.nlm.nih.gov/bioproject/PRJNA835901.
