# Supplementary material for: Mining candidate genes for rice cadmium accumulation in the shoot through a genome-wide association study and transcriptomic analysis
Source: Front Genet. 2022 Aug 31;13:944529. doi: 10.3389/fgene.2022.944529 (PMC9471252; doi:10.3389/fgene.2022.944529)
Supplement: Supplementary file 1 [file Presentation1.pptx]

## Slide 1
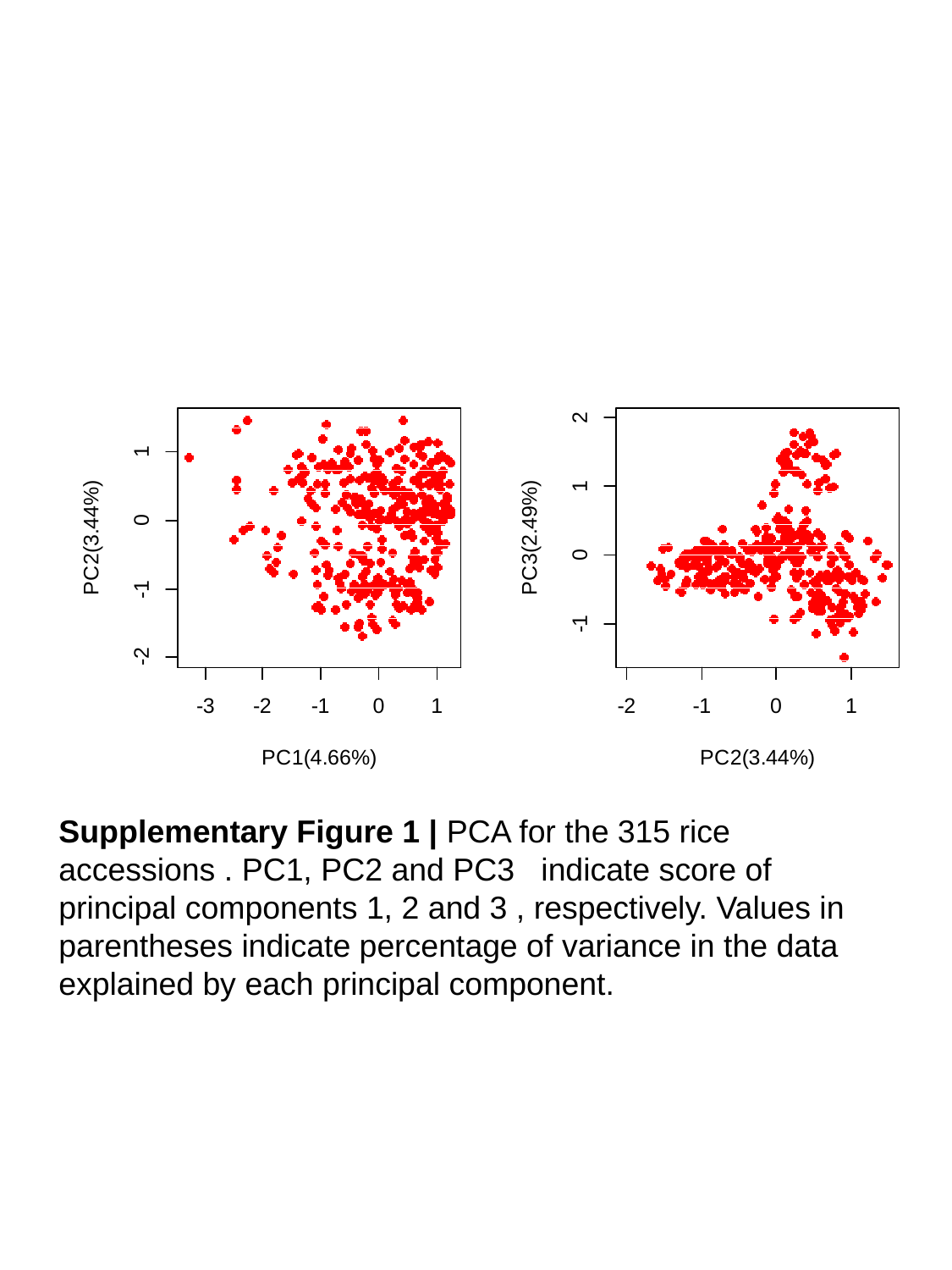

Supplementary Figure 1 | PCA for the 315 rice accessions . PC1, PC2 and PC3 indicate score of principal components 1, 2 and 3 , respectively. Values in parentheses indicate percentage of variance in the data explained by each principal component.

## Slide 2
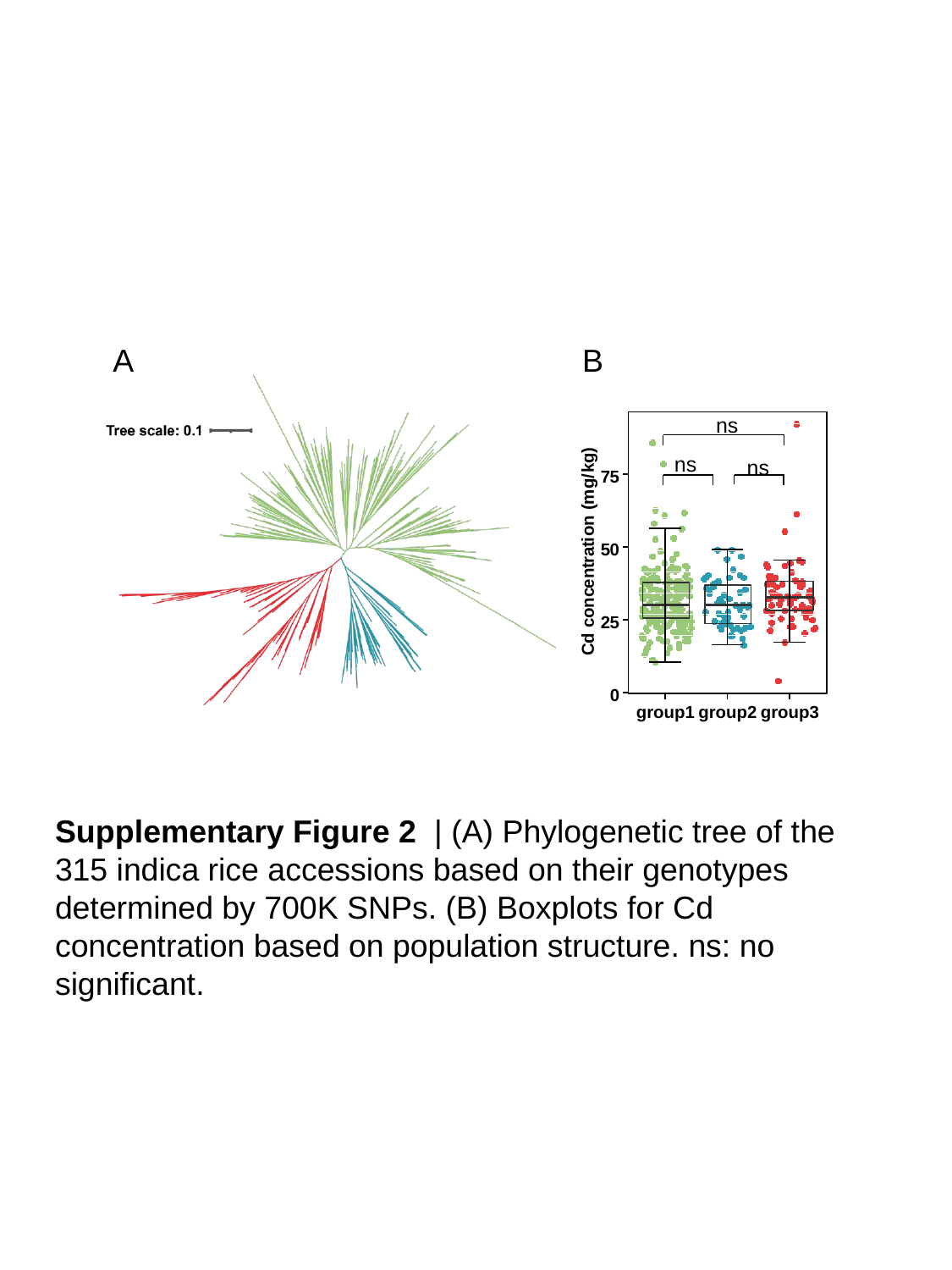

#
A
B
ns
ns
ns
Supplementary Figure 2 | (A) Phylogenetic tree of the 315 indica rice accessions based on their genotypes determined by 700K SNPs. (B) Boxplots for Cd concentration based on population structure. ns: no significant.

## Slide 3
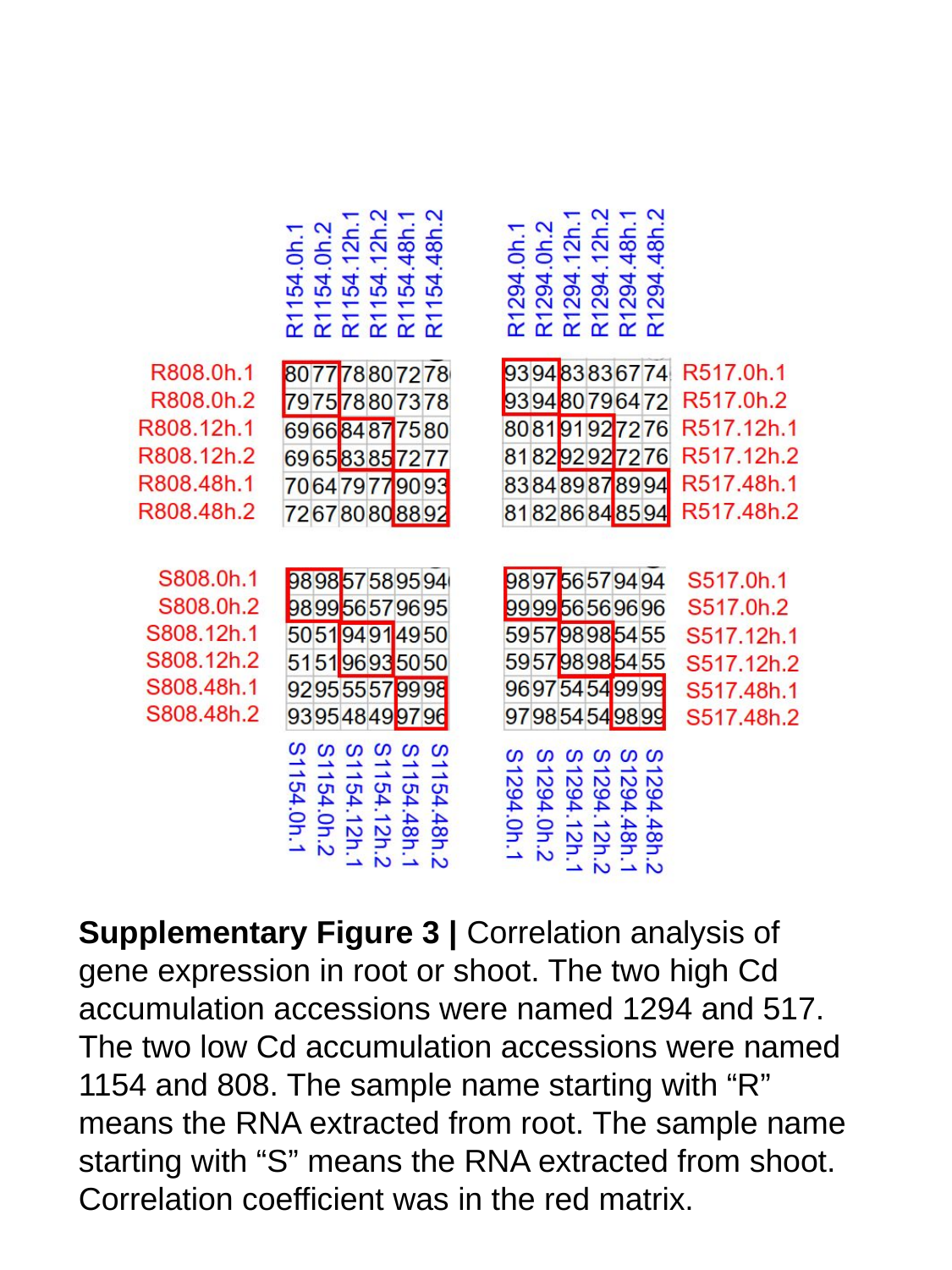

Supplementary Figure 3 | Correlation analysis of gene expression in root or shoot. The two high Cd accumulation accessions were named 1294 and 517. The two low Cd accumulation accessions were named 1154 and 808. The sample name starting with “R” means the RNA extracted from root. The sample name starting with “S” means the RNA extracted from shoot. Correlation coefficient was in the red matrix.

## Slide 4
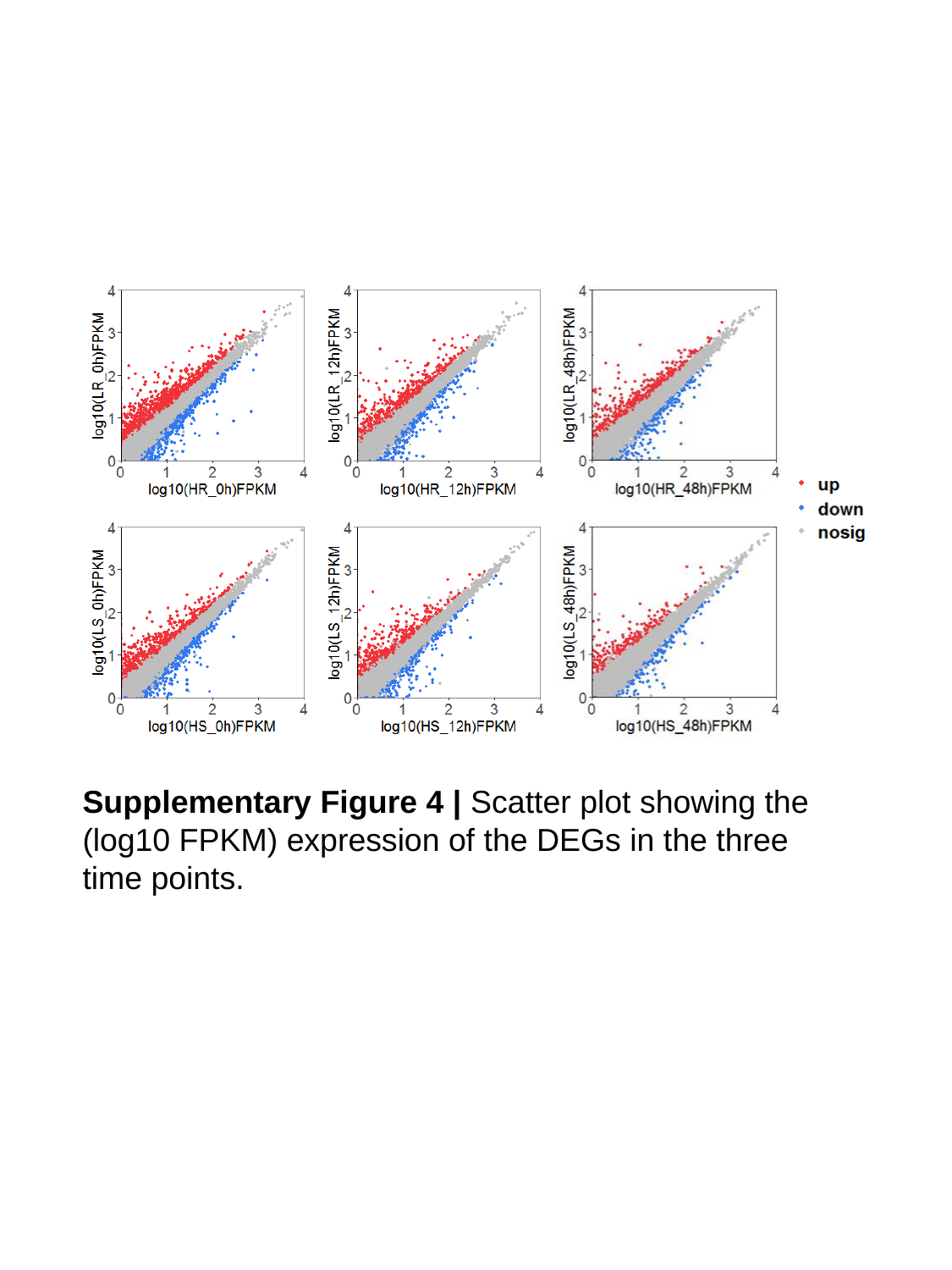

Supplementary Figure 4 | Scatter plot showing the (log10 FPKM) expression of the DEGs in the three time points.

## Slide 5
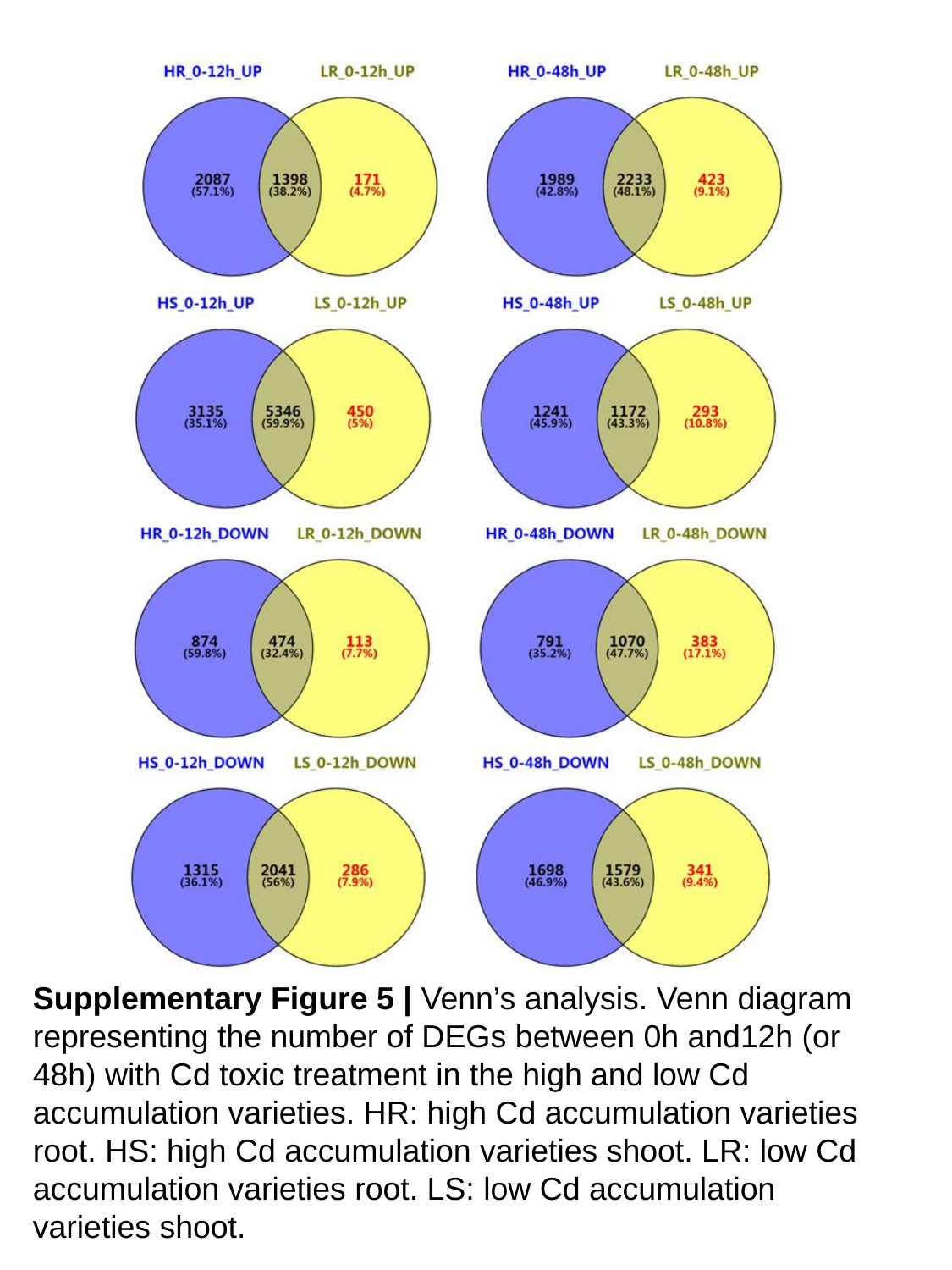

Supplementary Figure 5 | Venn’s analysis. Venn diagram representing the number of DEGs between 0h and12h (or 48h) with Cd toxic treatment in the high and low Cd accumulation varieties. HR: high Cd accumulation varieties root. HS: high Cd accumulation varieties shoot. LR: low Cd accumulation varieties root. LS: low Cd accumulation varieties shoot.
